# The Natural Antiangiogenic Compound AD0157 Induces Caspase-Dependent Apoptosis in Human Myeloid Leukemia Cells

**DOI:** 10.3389/fphar.2017.00802

**Published:** 2017-11-07

**Authors:** Melissa García-Caballero, Beatríz Martínez-Poveda, Miguel A. Medina, Ana R. Quesada

**Affiliations:** ^1^Departamento de Biología Molecular y Bioquímica, Facultad de Ciencias, Andalucía Tech, Universidad de Málaga, Málaga, Spain; ^2^Unidad 741 de CIBER “de Enfermedades Raras” (CIBERER), Málaga, Spain

**Keywords:** AD0157, myeloid leukemia, caspases, apoptosis, extrinsic/intrinsic pathways, mitochondria

## Abstract

Evasion of apoptosis is a hallmark of cancer especially relevant in the development and the appearance of leukemia drug resistance mechanisms. The development of new drugs that could trigger apoptosis in aggressive hematological malignancies, such as AML and CML, may be considered a promising antileukemic strategy. AD0157, a natural marine pyrrolidinedione, has already been described as a compound that inhibits angiogenesis by induction of apoptosis in endothelial cells. The crucial role played by defects in the apoptosis pathways in the pathogenesis, progression and response to conventional therapies of several forms of leukemia, moved us to analyze the effect of this compound on the growth and death of leukemia cells. In this work, human myeloid leukemia cells (HL60, U937 and KU812F) were treated with AD0157 ranging from 1 to 10 μM and an experimental battery was applied to evaluate its apoptogenic potential. We report here that AD0157 was highly effective to inhibit cell growth by promotion of apoptosis in human myeloid leukemia cells, and provide evidence of its mechanisms of action. The apoptogenic activity of AD0157 on leukemia cells was verified by an increased chromatin condensation and DNA fragmentation, and confirmed by an augmentation in the apoptotic subG1 population, translocation of the membrane phosphatidylserine from the inner face of the plasma membrane to the cell surface and by cleavage of the apoptosis substrates PARP and lamin-A. In addition, AD0157 in the low micromolar range significantly enhanced the activities of the initiator caspases-8 and -9, and the effector caspases-3/-7 in a dose-dependent manner. Results presented here throw light on the apoptogenic mechanism of action of AD0157, mediated through caspase-dependent cascades, with an especially relevant role played by mitochondria. Altogether, these results suggest the therapeutic potential of this compound for the treatment of human myeloid leukemia.

## Introduction

Leukemias are malignant neoplasms involving abnormally proliferating neoplastic cells that are originally derived from haematopoietic precursor cells and stem cells. These may in turn escape into the blood where they may be present in large numbers, resulting in the clinical presentations of the disease (Itzykson et al., 2017). Although recent advances in stem cell transplantation have brought new hope to sufferers of the disease, the treatment of leukemias is still mainly carried out with chemotherapy (Dombret and Gardin, 2016; Saygin and Carraway, 2017). Among the different types of leukemia that can be found in the patients, acute myeloid leukemia (AML) is the one presenting a highest prevalence in adults, and the age is the most prominent patient-specific risk factor (Liersch et al., 2014; Siegel et al., 2017). The overall 5-year survival for leukemia patients has more than quadrupled since 1960, reaching 70%. Nevertheless, in the case of AML this is still reduced to 25%, with more than 10000 estimated deaths for this type of leukemia in 2016, just in the USA (Siegel et al., 2017). In Europe, the estimated deaths in 2016 in all leukemia types were 23000 men and 19100 women (Malvezzi et al., 2016). Taking this data into account, the need for novel and more effective drugs to treat this disease is unquestionable.

Leukemia cells share some hallmarks with other tumor cells: they are self-sufficient in mitogenic/growth signals, they have limitless replication performance, they undergo sustained angiogenesis and they achieve tissue invasion and metastasis, among others (Hanahan and Weinberg, 2011). In addition, the evasion of apoptosis is another hallmark of cancer present in leukemia and other tumor cells (Vaux and Korsmeyer, 1999). Apoptosis, a physiological phenomenon that occurs throughout life during development and is initiated after cells are exposed to cytotoxic stresses including UV irradiation, hypoxia, serum deprivation and drugs, is crucial for the development and homeostasis of hematopoiesis (Pistritto et al., 2016). Deregulated apoptosis plays a relevant role in the outcome of leukemia cells and in the appearance of resistance mechanisms to antitumor drugs, which compromise the success of the chemotherapy currently used in clinics (Reed and Pellecchia, 2005; Testa and Riccioni, 2007; Fulda, 2009; Lück et al., 2011; Vogler et al., 2017). In the last decade, the induction of apoptotic cell death is attracting attention as a new strategy to kill cancer cells, being considered a novel target for cancer chemoprevention and a new strategy to increase the responsiveness of human cancer toward the conventional therapies used in patients (Sun et al., 2004; Kim et al., 2006; Fulda, 2009; Hassan et al., 2014). Thus, the development of new drugs that are able to trigger apoptosis in aggressive hematological malignancies, such as AML and chronic myeloid leukemia (CML), may be considered a promising antileukemic strategy.

Most apoptosis signaling pathways cause the activation of a cascade of caspases (cysteine-aspartic proteases) and endonucleases responsible for the cleavage of cellular proteins and DNA, giving rise to plasma membrane blebbing, cell shrinkage, apoptotic body formation, chromatin condensation and DNA fragmentation (Vermeulen et al., 2005). Caspases are classified into 2 groups, initiator and executioner caspases, according to their level of action. They are implicated in two main signaling pathways controlling apoptosis, termed extrinsic and intrinsic pathways and started by different initiator caspases (Man and Kanneganti, 2016). The extrinsic pathway, a death receptor dependent cascade, involves the binding of extracellular death ligands, such as TNF ligand or TRAIL, to death receptors, provoking the recruitment of adaptor protein (FADD), which also interacts with procaspase-8 and -10 to form the death-inducing signaling complex (DISC) (Kim et al., 2006). Once activated, caspase-8 triggers a caspase cascade that bypasses mitochondria, leading directly to cell death by activating downstream executioner caspases, such as caspase-3 and -7 (Hui et al., 2011). The intrinsic pathway, a receptor independent cascade, involves the loss of mitochondrial membrane potential, the release of cytochrome c and the subsequent activation of caspase-9. Both caspases-8 and -9 are considered initiator caspases and their activation leads to the activation of downstream executioner caspases, responsible for the culmination of cell death (Lee et al., 2012; Reubold and Eschenburg, 2012; Man and Kanneganti, 2016). Finally, an increasing number of non-caspase-mediated cell death pathways are becoming known (Pradelli et al., 2010; Vanden Berghe et al., 2014).

A large proportion of the currently used or still undergoing clinical trials anticancer drugs, are derived from natural sources (Amirkia and Heinrich, 2015). Most of them have been isolated from plants and terrestrial microorganisms, mainly due to their higher availability and as a result of their therapeutic effects had been previously known in traditional medicines (Kinghorn et al., 2010). Although still mostly unexplored, marine organisms have a high potential as sources of new interesting and singular pharmacological lead compounds, derived from the large diversity of marine habitats and environmental conditions. As an adaptation mechanism to survive in extreme environments, marine organisms have developed chemical means of defense that include the production of toxic metabolites, yielding an increasing number of products of the highest interest for drug discovery (Blunt et al., 2017). Interestingly, the Food and Drug Administration (FDA) have already approved several marine compounds as anticancer drugs (Malve, 2016).

**FIGURE 1:**
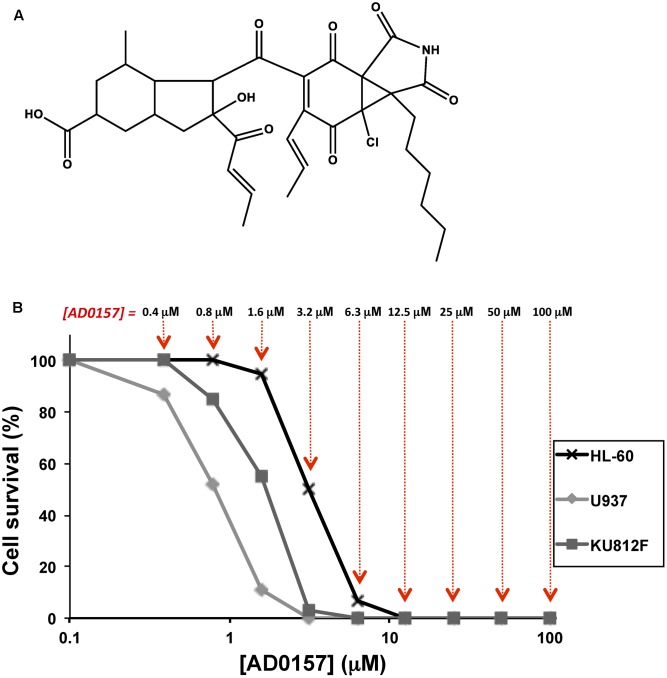
AD0157 inhibits the growth of human myeloid leukemia cells. **(A)** Chemical structure of AD0157. **(B)** Representative survival curves with the dose-dependent effect of AD0157 on the *in vitro* growth of HL60 (x), U937 (ding117) and KU812F (aaa). Cell survival is represented as a percentage of control-cell growth in cultures containing no drug. Each point represents the mean of quadruplicates; SD values were typically lower than 10% of the mean values and are omitted for clarity. The different AD0157 concentrations are displayed at the top.

AD0157, a pyrrolidinedione (**Figure 1A**) isolated from the fermentation broth of the marine fungus *Paraconiothyrium* sp. HL-78-gCHSP3-B005, has been previously described by us as a potent inhibitor of angiogenesis *in vitro*, *ex vivo*, and *in vivo* (García-Caballero et al., 2014). The antiangiogenic activity of AD0157 seems to be due, at least in part, to the induction of apoptosis in activated endothelial cells. The crucial role played by defects in the apoptosis pathways in the pathogenesis, progression and response to conventional therapies of several forms of leukemia, moved us to analyze the effect of this compound on the growth and death of leukemia cells. Therefore, the purpose of our study was to evaluate whether AD0157 could inhibit the growth and induce caspase-dependent apoptosis in three human myeloid leukemia cell lines, determining its therapeutic potential for the treatment of myeloid leukemia.

## Materials and Methods

### Materials

Cell culture media, penicillin, streptomycin and amphotericin B were purchased from Biowhittaker (Walkersville, MD, United States). Fetal bovine serum (FBS) was a product of Harlan-Seralab (Belton, United Kingdom). Plastics for cell culture were supplied by NUNC (Roskilde, Denmark). AD0157 was isolated and purified from the fermentation broth of a marine fungus by Instituto Biomar (León, Spain). It was dissolved in dimethyl sulfoxide (DMSO) at a concentration of 20 mM and stored at -20°C until use. Antibodies were obtained from Cell Signaling Technology (Danvers, MA, United States), Santa Cruz Biotechnology (Dallas, TX, United States) and BD Biosciences (Bedford, MA, United States). Supplements and other chemicals not listed in this section were obtained from Sigma Chemicals Co. (St. Louis, MO, United States).

### Cell Cultures

Human myeloid leukemia cell lines: HL60 (promyelocytic leukemia), U937 (histiocytic lymphoma), both of them AML cell lines, and the CML KU812F cell line (basophilic leukemia), were purchased from American Type Culture Collection (ATCC, Manassas, VA, United States). HL60, U937 and KU812F cell lines were cultured in RPMI-1640 medium containing 2 mM glutamine, 50 U/ml penicillin, 50 μg/mL streptomycin, 1.25 μg/mL amphotericin B and supplemented with 20% heat inactivated FBS in the case of HL60 cell line, or with 10% heat inactivated FBS in the case of U937 and KU812F cell lines. Cells were maintained at 37°C in a humidified 5% CO_2_ atmosphere. Cells were treated with AD0157 (1, 5, and 10 μM) or with the vehicle control (0.05% DMSO) in the different approaches.

### Cell Growth Assay

For cell proliferation tests, HL60, U937 or KU812F cells (2 × 10^3^ cells/well) were incubated in serial dilutions of AD0157, in a final volume of 100 μL of their respective complete medium. After 3 days of incubation (37°C and 5% CO_2_ in a humid atmosphere), the cell growth was analyzed using the 3-(4,5-dimethylthiazol-2-yl)-2,5-diphenyltetrazolium bromide or MTT dye reduction assay (García-Caballero et al., 2014), as follows: 10 μL of MTT (5 mg/mL in phosphate-buffered saline or PBS) was added to each well and the plate was incubated for a further 4 h at 37°C. This assay is dependent on the reduction of MTT by mitochondrial dehydrogenases of viable cell to a blue formazan product, which can be dissolved in 150 μL of 0.04 N HCl/2-propanol and read spectrophotometrically at 550 nm. All determinations were carried out in quadruplicate and three independent experiments were performed. IC_50_ values were calculated as those concentrations of AD0157 yielding 50% of cell survival, taking the values obtained for untreated cells as 100%.

### Hoechst 33258 Staining and Study of Nuclear Morphology

The study of nuclear morphologic changes induced by AD0157 was assessed by Hoechst 33258 stainings (García-Caballero et al., 2011). Thus, 5 × 10^5^ human myeloid leukemia cells/well were seeded in complete medium on 8-well Falcon humidified chamber slides and incubated at 37°C in a humidified 5% CO_2_ atmosphere, with or without the indicated concentrations of AD0157 for 14 h. After incubation, cells were washed with PBS, fixed with formalin solution and stained with Hoechst 33258 (1 μg/mL in PBS), using a cytospin. Cells were mounted on slides using DAKO Cytomation Fluorescent Mounting Medium (DAKO, Denmark) and observed under a fluorescence microscope (Leica, TCS-NT, Heidelberg, Germany).

### DNA Fragmentation Analysis

For DNA fragmentation study, human myeloid leukemia cells (5 × 10^5^ cells per well) were seeded in complete medium on 8-well Falcon humidified chamber slides and incubated for 14 h in presence or absence of different doses of the studied compound, as previously described by Crowley et al. (2016). After incubation, cells were washed with PBS, fixed with formalin solution, washed with PBS and permeabilized with 0.1% Triton X-100 in PBS, using a cytospin. The TUNEL (terminal deoxynucleotidyl transferase mediated dUTP-biotin nick end-labeling) assay was performed with the use of the In Situ Cell Death Detection Kit (Roche Diagnostics, Barcelona, Spain), according to the manufacturer’s instructions. Finally, after cell mounting on slides, samples were visualized under a fluorescence microscope.

### Cell Cycle Studies

Human myeloid leukemia cells were assessed by flow cytometric analysis using propidium iodide-stained cells (García-Caballero et al., 2013). Firstly, 2 × 10^6^ cells were incubated in 6-well plates in presence or absence of the indicated concentrations of AD0157. After 14 h treatment, cells were washed with PBS and fixed with 70% ethanol for 1 h on ice. Pelleted cells were incubated with RNase-A (0.1 mg/mL) and propidium iodide (40 μg/mL) for 1 h with shaking and protected from light. Percentages of subG1, G1, S/G2/M populations were determined using a MoFlo Dakocytomation cytometer (Dako, Denmark) and the software Summit 4.3. In some experiments, either the caspase-8 selective inhibitor Z-IETD-FMK, the caspase-9 selective inhibitor Z-LEHD-FMK or the pan inhibitor of caspases Z-VAD-FMK at 50 μM were used (BD Biosciences, Madrid, Spain). Cells were pre-treated with the caspase inhibitor for 2 h prior to treatment with AD0157 (14 h) and the percentage of subG1 population was determined by flow cytometry.

### PE-Annexin V and 7-Aminoactinomycin D Stainings

Apoptosis was examined by flow cytometry using the PE-Annexin V apoptosis kit (Pharmingen, BD Biosciences, San Agustin de Guadalix, Spain) as previously described by Martínez-Poveda et al. (2012). Human myeloid leukemia cells were seeded in 6-well plates (2 × 10^6^ cells/well) in complete growth medium with or without the studied compound. After 14 h incubation, cells were washed with PBS and stained with phycoerythrin (PE)-labeled Annexin V and 7-aminoactinomycin D (7AAD), following the manufacturer’s instructions. Samples were analyzed by using the MoFlo Dakocytomation cytometer (Dako, Denmark) and the different cell populations were evaluated with the software Summit 4.3. The 7AAD-/PE-Annexin V-, 7AAD-/PE-Annexin V+, 7AAD+/PE-Annexin V+ and 7AAD +/PE-Annexin V- cell populations, correspond to viable, early apoptotic, late apoptotic and necrotic cells, respectively.

### Measurement of the Mitochondrial Membrane Potential (ΔΨm)

The mitochondrial membrane potential (ΔΨm) was assessed by the retention of Rhodamine 123, a membrane-permeable fluorescent cationic dye that is selectively taken up by mitochondria (Baracca et al., 2003). In brief, cells (1 × 10^6^) were plated in 6-well plates, cultured with or without AD0157 for 14 h and incubated with 0.1 μg/mL Rhodamine 123 in the dark for 30 min at room temperature, prior to termination of the experiment. Subsequently, cells were collected, washed with PBS and analyzed by flow cytometry, with excitation and emission wavelengths of 495 and 535 nm, respectively. The levels of Rhodamine 123 retention in mitochondria were proportional to the ΔΨm.

### Caspase Activity Assay

The determination of caspase-8, -9 and -3/-7 activities were carried out after leukemia cell treatment with AD0157 or vehicle. The times of incubation with the studied compound were 6 h, 30 min and 14 h for caspase-8, -9 and -3/-7, respectively. Then, Caspase-Glo^®^ 8, Caspase-Glo^®^ 9 and Caspase-Glo^®^ 3/7 reagents (Promega Biotech Ibérica, Madrid, Spain), were added to wells, according to the manufacturer’s instructions and the luminescence was recorded after 30 min with a GLOMAX 96 microplate luminometer (Promega Biotech Ibérica) and the software “Glomax 1.9.0.” This assay provides a proluminescent caspase-8 Z-LETD-aminoluciferin substrate, a proluminescent caspase 9 Z-LEHD-aminoluciferin or a proluminescent caspase-3/-7 Z-DEVD-aminoluciferin substrate, and a proprietary thermostable luciferase in a reagent optimized for caspase-8, -9 and -3/-7 activity, luciferase activity and cell lysis.

### Detection of Cytochrome c Release in Permeabilized Cells

Human myeloid leukemia cells were incubated with AD0157 for 14 h in 6-well plates and samples were further analyzed as described in Martínez-Poveda et al. (2012). After washing with PBS, 2 × 10^6^ cells were suspended in 100 μl of assay buffer (MSH buffer - 210 mM mannitol, 70 mM sucrose, 5 mM HEPES, pH 7.5- supplemented with 50 mM KCl, 1 mM EGTA, 5 mM succinate and 5 mM MgCl_2_), permeabilized with 10 μg of cold digitonin for 5 min at room temperature, and centrifuged at 13000 rpm for 5 min. The supernatants containing soluble cytosolic fraction, as well as the pellets containing mitochondria, were mixed with Laemmli’s buffer. Finally, the samples were boiled for 5 min at 95°C and cytochrome c release was analyzed by Western blot.

### Western Blot Analyses

Cell cultures (2 × 10^6^ cells/well) were incubated in RPMI medium supplemented with 5% FBS and AD0157 for 14 h. Then, cells were stimulated for 30 min with medium containing 20% FBS and harvested for analyses. The protein lysates were obtained by scrapping the cells in a lysis buffer (50 mM Tris, pH 7.4, 150 mM NaCl, 1% Triton X-100, 0.25% sodium deoxycholate, 1 mM EDTA, 1 mM sodium orthovanadate and 5 mg/mL of a protease inhibitors mixture). Afterward, extracts were centrifuged at 13000 rpm for 15 min at 4°C, evaluated for protein concentration and stored at -80°C until the moment of analysis. These samples were mixed with Laemmli’s buffer, denatured for 5 min at 95°C and submitted to SDS-PAGE. After electrophoresis, samples were electrotransferred to nitrocellulose membranes, blocked with 5% dried skimmed milk in 50 mM Tris pH 8.4, 0.9% NaCl, 0.05% Tween 20 (Tris buffered saline-Tween 20, TBS-T), and incubated overnight in the presence of different antibodies: anti-human cleaved lamin-A, anti-human total and cleaved PARP, anti-human total and phosphorylated Bad (Ser112), anti-human total and phosphorylated Akt (Ser473), anti-human total and phosphorylated ERK1/2 (p44/42 MAPK) (all of them from Cell Signaling Technology) at a dilution of 1:1000 in TBS-T with 5% BSA or non-fat dry milk. The anti-human cytochrome c (BD Biosciences) was used at a dilution of 1:250 in TBS-T with 5% BSA. After three washing steps with TBS-T, the membranes were incubated with horseradish peroxidase-conjugated anti-rabbit or anti-mouse secondary antibodies (Cell Signaling Technology) at a dilution of 1:2000 in blocking buffer for 2 h at room temperature. After three washing steps with TBS-T, the immunoreactive bands were detected using a chemiluminescence system (SuperSignal West Pico Chemiluminescent Substrate, Pierce, Rockford, IL, United States) with an imaging system (Chemidoc XRS System, Bio-Rad, Hercules, CA, United States) and were quantified by using ImageLab version 3.0 software. The membranes were incubated with an anti-GAPDH primary antibody at a dilution of 1:1000 to ensure equal loading. Phosphorylation inhibition was calculated as the phosphorylated protein/total protein ratio. All bands were compared with their controls and expressed as means ± SD of 3 independent experiments.

### Statistical Analysis

All data are expressed as means ± standard deviation (SD) of three independent experiments. Two-tailed Student’s *t*-test was used for evaluations of pairs of means, to establish which groups differed from the control group. *p* < 0.05 was considered to be statistically significant.

## Results

### AD0157 Inhibits the Growth of Human Myeloid Leukemia Cells

To elucidate the effect of AD0157 on the growth of human leukemia cells, MTT assays were performed using two human AML cell lines (HL60 and U937) and the Philadelphia-positive CML KU812F cell line (Koeffler and Golde, 1980; Larrick et al., 1980; Fukuda et al., 1987). Logarithmically proliferating cells were treated with different concentrations of AD0157 (from 100 to 0.4 μM) for 72 h. In **Figure 1B**, representative dose-response curves are displayed and each point represents the mean of quadruplicates. SD values were typically lower than 10% of the mean values and were omitted for clarity. The half-maximal inhibitory concentration (IC_50_) value was calculated from dose-response curves as the concentration of compound yielding 50% of control cell survival. AD0157 inhibited cell growth in a dose-dependent manner and the IC_50_ values of this effect were 2.67 ± 0.76 μM for HL60, 0.70 ± 0.26 μM for U937 and 1.80 ± 0.63 μM for KU812F (means ± SD of 3 independent experiments with quadruplicate samples each).

### AD0157 Induces Chromatin Condensation and DNA Fragmentation in Human Myeloid Leukemia Cells

**FIGURE 2:**
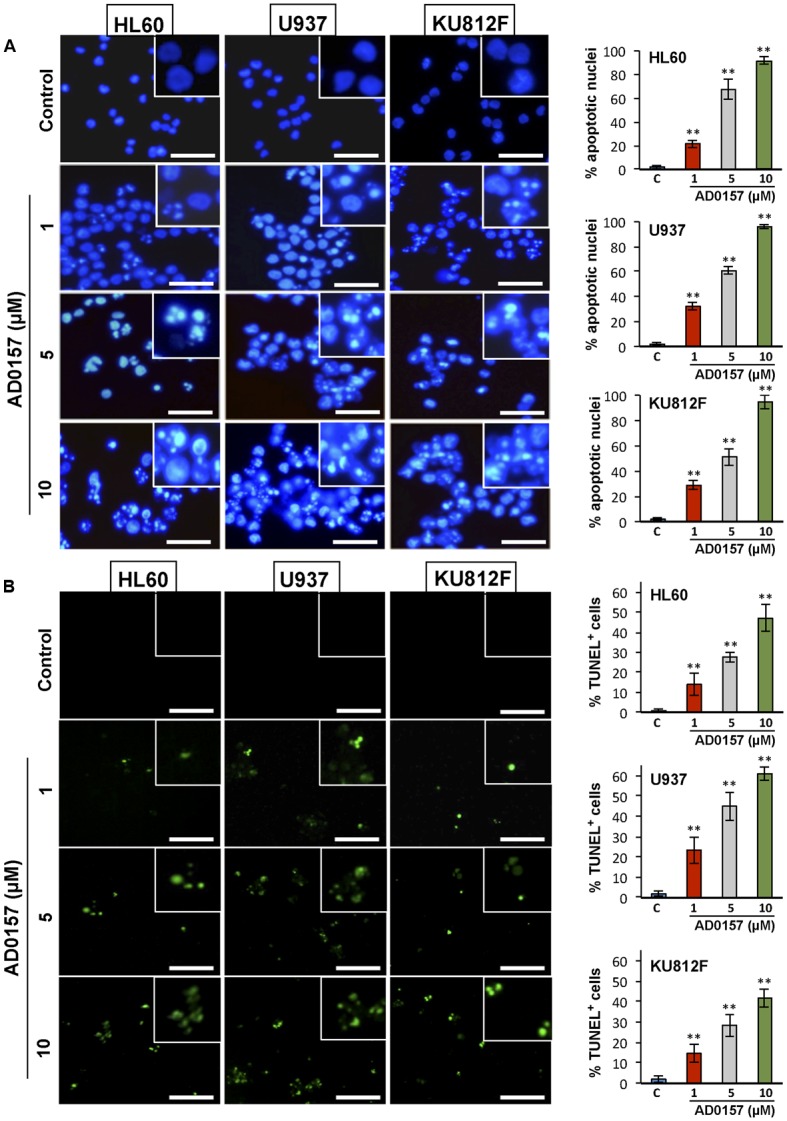
AD0157 induces chromatin condensation and DNA fragmentation in HL60, U937 and KU812F cells. **(A)** Effect of AD0157 on human myeloid cell morphology and chromatin condensation evaluated by Hoechst staining (bar = 50 μm). Graphs represent the percentage of control and AD0157-treated cells showing condensed chromatin. Values are expressed as means ± SD of the counts evaluated in ten vision fields. ^∗∗^*p* < 0.001 *versus* untreated cells. **(B)** Effect of AD0157 on DNA fragmentation assessed by TUNEL labeling (bar = 50 μm). Graphs represent the percentage of control and AD0157-treated showing TUNEL positivity. Values are expressed as means ± SD of the counts evaluated in ten vision fields. ^∗∗^*p* < 0.001 *versus* untreated cells.

To further examine whether AD0157 was able to induce apoptosis in human leukemia cells, a first pilot study with different doses of AD0157 was carried out. Three of these AD0157 concentrations (1, 5, and 10 μM) were selected to show a dose-dependent effect after 14 of treatment with AD0157. In the first approach, leukemia cells were exposed to AD0157 for 14 h and their DNA stained with Hoechst 33258. As shown in **Figure 2A**, AD0157 at a concentration as low as 1 μM induced nuclear chromatin condensation and cell shrinkage in the three studied cell lines. This effect was dose-dependent, since at AD0157 5 μM the number of apoptotic cells was higher and, after incubation with 10 μM of compound, most of nuclei exhibited condensed chromatin. Additionally, DNA fragmentation was detected with the terminal deoxynucleotidyl transferase-mediated dUTP-biotin nick end-labeling (TUNEL) assay, and AD0157-treated cells at doses of 1, 5, and 10 μM revealed fluorescent nuclei as a result of DNA damage (**Figure 2B**).

### SubG1 Population Is Increased in AD0157-Treated Human Myeloid Leukemia Cells

**FIGURE 3:**
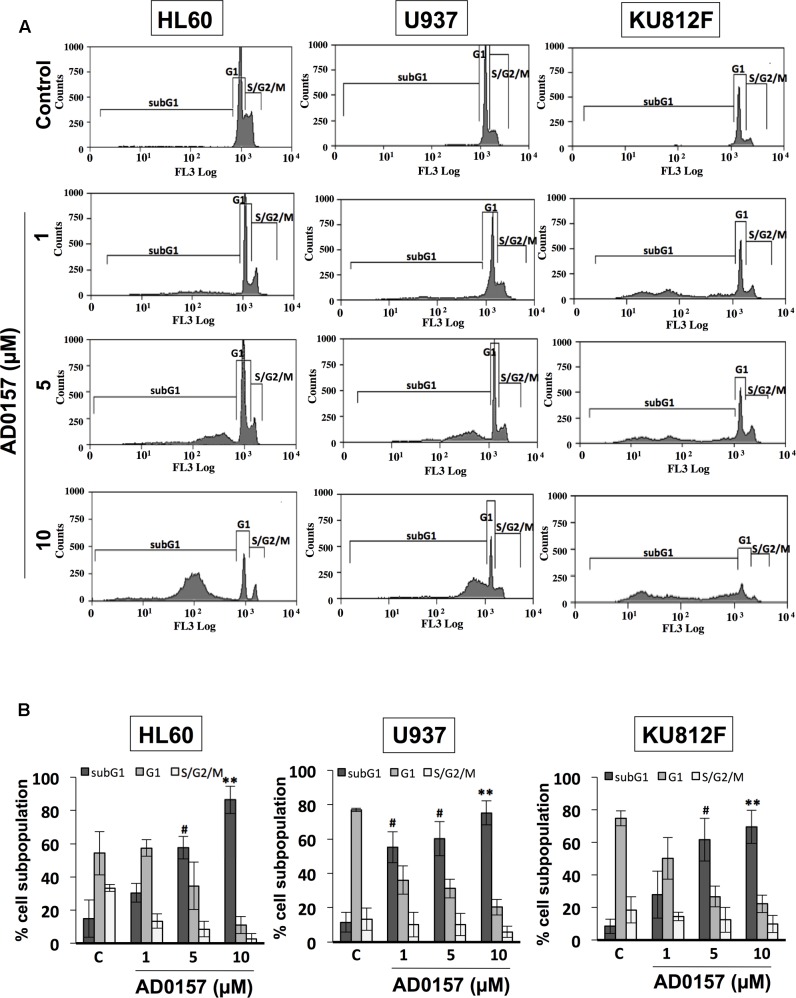
AD0157 induces increases in the subG1 population of myeloid leukemia cells. **(A)** Representative flow cytometry histograms showing the effect of AD017 on HL60, U937 and KU812F cell cycle distribution. Leukemia cells were exposed for 14 h to AD0157 at the indicated concentrations, stained with propidium iodide and percentages of subG1, G1, and S/G2/M cells were determined using a MoFlo DakoCytomation cytometer. **(B)** Graphs correspond to the distribution of cell subpopulation percentages expressed as means ± SD of three independent experiments. ^#^*p* < 0.005 and ^∗∗^*p* < 0.001 *versus* untreated cells.

In order to determine whether alterations in the cell cycle distribution were responsible for AD0157-mediated cell growth inhibition and apoptosis induction, the human leukemia cell subpopulations in the different phases of cell cycle were quantified by flow cytometry upon AD0157 treatment (1, 5, and 10 μM). When cells were treated with the compound for 14 h and then, stained with propidium iodide, the subG1 population representing the apoptotic cells with subdiploid DNA content, suffered an increase with a dose-dependent pattern as compared to untreated cells in the evaluated cell lines (**Figure 3A**). The percentage of cells in subG1 phase was significantly increased upon treatment with 5 and 10 μM of compound. Indeed, the subG1 percentages in 5 μM AD0157-treated *versus* untreated control cells were 57.5% *versus* 14.9% (for HL-60), 60.2% *versus* 11.5% (for U937) and 61.6% *versus* 8.4% (for KU812F), (*p* < 0.005), respectively (**Figure 3B**). These data suggest that induction of apoptosis, rather than inhibition of cell proliferation, could be a major mechanism of the observed leukemia cell growth inhibition by AD0157.

### AD0157 Induced Early and Late Apoptosis and Cleavage of Lamin-A and PARP in Human Myeloid Leukemia Cells

**FIGURE 4:**
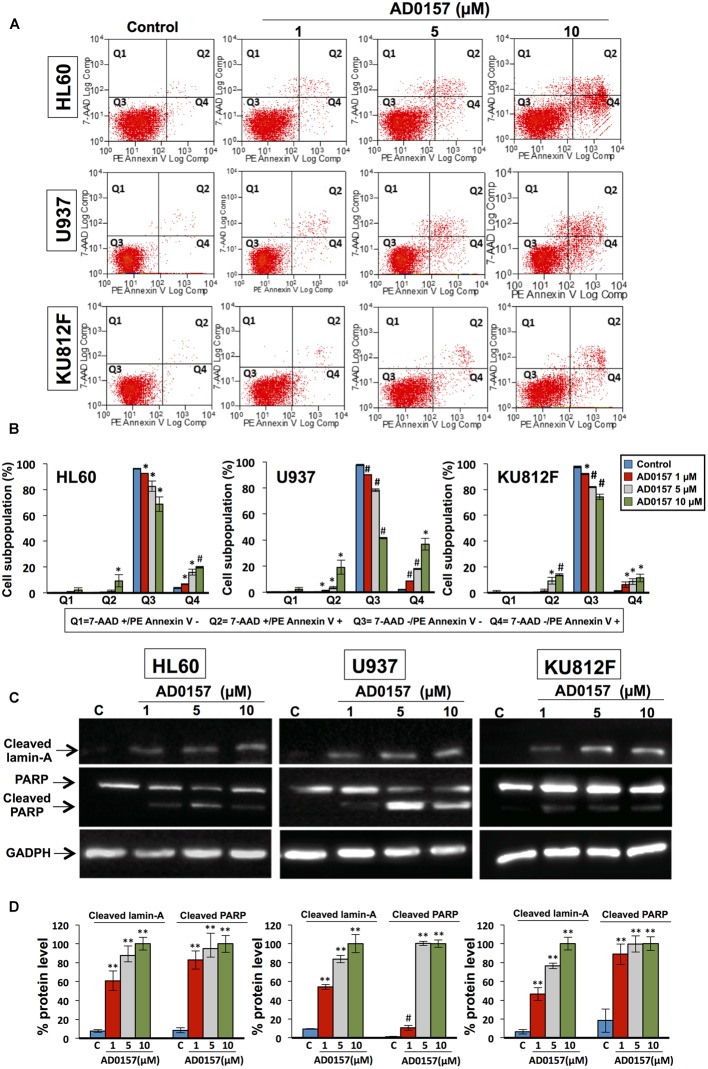
AD0157 causes early and late apoptosis and cleavage of lamin-A and PARP in human myeloid leukemia cells. **(A)** Representative examples of flow cytometry dot blots for untreated and AD0157-treated leukemia cells. Q1: 7AAD+/PE-Annexin V- (necrotic cells), Q2: 7AAD+/PE-Annexin V+ (late apoptotic cells), Q3: 7AAD -/PE-Annexin V- (viable cells) and Q4: 7AAD-/PE-Annexin V+ (early apoptotic cells). HL60, U937 and KU812F cells were incubated with AD0157 at the indicated concentrations for 14 h and assessed by 7AAD/PE-Annexin V stainings and flow cytometry studies. **(B)** Quantification of the different populations (necrotic, late and early apoptotic and viable leukemic cells). Values are expressed as means ± SD of three independent experiments. ^∗^*p* < 0.05 and ^#^*p* < 0.005 *versus* control. **(C)** Western blots of cleaved lamin-A, PARP and cleaved PARP in HL60, U937 and KU812F control cells and treated for 14 h with the indicated concentrations of AD0157. **(D)** Quantification by densitometry of Western blot bands. Results are expressed as the average relative protein level percentage ± SD of three independent experiments. The percentage obtained with the higher dose of compound is considered the 100%. ^#^*p* < 0.005 and ^∗∗^*p* < 0.001 *versus* control.

After 14 h incubation with different doses of compound (1, 5, and 10 μM), the translocation of phosphatidylserine from the inner face of the phospholipid bilayer to the cell surface through PE-Annexin V and 7AAD stainings was analyzed by flow cytometry. As depicted in **Figure 4A**, AD0157 induced a significant augmentation in the percentage of both early (located in Q4) and late apoptotic (located in Q2) cell subpopulations, whereas no increases in the necrotic cell subpopulation (in Q1) were observed. Upon treatment with AD0157 10 μM, the cellular percentages in the Q4 and Q2 quadrants were 19.8 and 9.4%, 36.9 and 19.1%, and 11.4 and 13.8% for HL60, U937 and KU812F (*p* < 0.05 and *p* < 0.005), respectively (**Figure 4B**). Interestingly, the percentage of early apoptotic cells was higher in the AML cell lines than in the CML cell line.

In order to illustrate the molecular basis of the apoptosis induction in human leukemia cells by AD0157, the cleavage of lamin-A and poly (ADP-ribose) polymerase (PARP) was studied upon treatment with AD0157 at 1, 5, and 10 μM. Lamin-A, a nuclear membrane structural component belonging to the intermediate filament family, contributes to the scaffolding of the nuclear envelope and the maintenance of normal cell functions such as cell cycle control, DNA replication and chromatin organization (Gruenbaum et al., 2000). During apoptosis, lamin-A is specifically cleaved to a large (40–45 kDa) and a small (28 kDa) fragment, resulting in nuclear dysregulation and cell death. Western blot images and analyses showed that cleaved lamin-A bands were observed after treating leukemia cells with AD0157 at concentrations of 1 μM or higher (**Figures 4C,D**). On the other hand, PARP is a 116 kDa nuclear polymerase involved in DNA repair in response to environmental stress, helping cells to maintain their viability (Satoh and Lindahl, 1992). PARP cleavage by activated caspase-3 facilitates cellular disassembly and is used as an early marker for apoptosis induction. In human PARP, the cleavage separates the PARP amino-terminal DNA binding domain (24 kDa) from the carboxy-terminal catalytic domain (89 kDa). As shown in **Figures 4C,D**, PARP was cleaved to the 89 kDa proteolytic product upon treatment with AD0157 in the micromolar range. By contrast, no cleaved lamin-A and PARP proteins were detected in untreated cells.

### Human Myeloid Leukemia Cell Initiator and Effector Caspases Are Activated by AD0157

**FIGURE 5:**
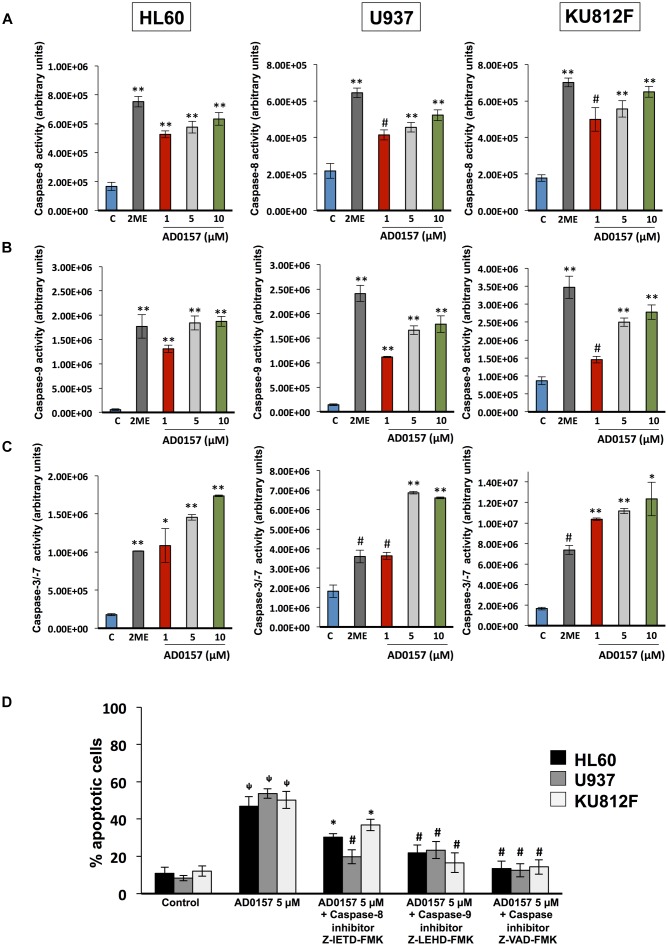
Caspase activation by AD0157 in human myeloid leukemia cells. **(A)** Effect of AD0157 on caspase-8, **(B)** caspase-9 and **(C)** caspase-3, -7 in HL60, U937 and KU812F. The determination of caspase-8, -9 and -3/-7 activities were carried out after leukemia cell treatment with AD0157 or vehicle for 6 h, 30 min and 14 h, respectively. Afterward, Caspase-Glo^®^ 8, Caspase-Glo^®^ 9 and Caspase-Glo^®^ 3/7 reagents were added and the luminescence was recorded at 30 min. Treatment with 10 μM 2-methoxyestradiol (2ME) was used as a positive control. Data of caspase activities are expressed in arbitrary units ± SD of three independent assays with 3 replicates each one. ^∗^*p* < 0.05, ^#^*p* < 0.005, and ^∗∗^*p* < 0.001 *versus* control. **(D)** Percentage of subG1 population (apoptotic cells) after pre-treatment for 2 h with either caspase-8 inhibitor Z-IETD-FMK, caspase-9 inhibitor Z-LEHD-FMK or pan caspase inhibitor Z-VAD-FMK, followed by AD0157 5 μM treatment for 14 h and flow cytometric analyses. Data are expressed as means ± SD of three independent experiments. ^Ψ^*P* < 0.005 *versus* untreated controls. ^∗^*p* < 0.05 and ^#^*p* < 0.005 *versus* cells treated with AD0157 5 μM in absence of caspase inhibitors.

To obtain greater insight into the biochemical mechanism used by AD0157 to induce apoptosis in human leukemia cells, caspase activities were investigated by using specific pro-luminescent substrates of caspase-8, -9 and -3/-7. The incubation with AD0157 at concentrations of 1 μM or higher (5 and 10 μM), significantly enhanced the activities of the initiator caspase-8 (**Figure 5A**) and caspase-9 (**Figure 5B**), and the effector caspases-3/-7 in a dose-dependent manner (**Figure 5C**) in the different leukemia cells. A positive control of caspase activation, 10 μM 2-methoxyestradiol, was used in these experiments. To confirm that apoptosis induced by AD0157 was a caspase-dependent mechanism we examined the effects of selective inhibitors of caspase-8 (Z-IETD-FMK) and caspase-9 (Z-LEHD-FMK), as well as those of a broad-spectrum caspase inhibitor (Z-VAD-FMK). Thus, pre-treated leukemia cells with these caspase inhibitors and treated with AD0157, were stained with propidium iodide and analyzed by flow cytometry. **Figure 5D** shows that the apoptogenic activity of AD0157, represented by the percentage of cells in subG1 phase, was fully blocked by addition of the pan-caspase inhibitor Z-VAD-FMK, with significant partial reversal of AD0157-induced apoptosis being obtained with the caspase-8 and caspase-9 inhibitors (Z-IETD-FMK and Z-LEHD-FMK, respectively). All the above suggest that both, intrinsic and extrinsic pathways activation, could be involved in the apoptogenic activity of AD0157.

### Mitochondria Is Involved in the Apoptogenic Activity of AD0157

**FIGURE 6:**
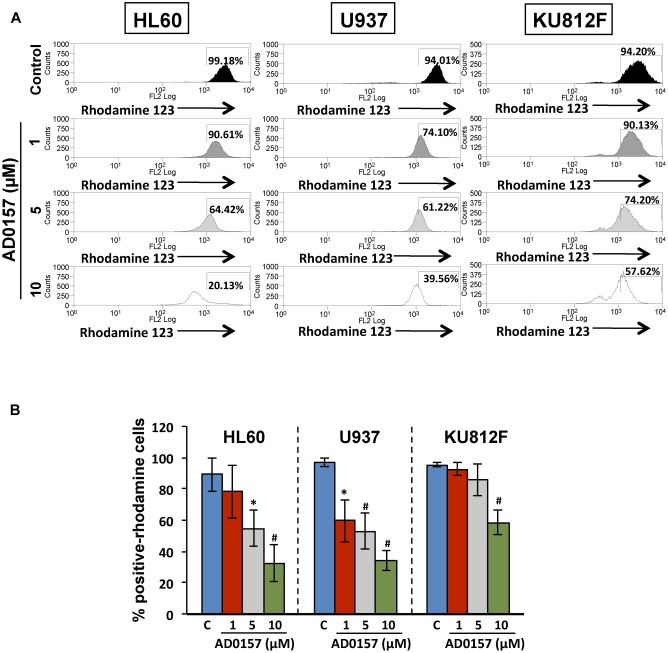
Mitochondrial membrane potential is involved in the apoptogenic activity of AD0157. **(A)** Flow cytometry histograms with the relative fluorescence intensity of human leukemia cells treated or not with AD0157 followed by Rhodamine 123 incubation. AD0157 decreases the Rhodamine 123 uptake in HL60, U937 and KU812 cells due to the mitochondrial membrane potential loss. Percentage of Rhodamine 123-positive cells is indicated in the histograms. **(B)** Quantification of Rhodamine 123-positive cell percentages, expressed as means ± SD from three independent experiments. ^∗^*p* < 0.05 and ^#^*p* < 0.005 *versus* control.

The membrane permeability transition of mitochondria is controlled by the mitochondrial membrane potential, represented as ΔΨm and playing a central role in the mitochondrial-mediated apoptosis and the caspase-9 activation. As can be observed in **Figure 6A**, in absence of AD0157, human leukemia cells analyzed by flow cytometry exhibited an intact ΔΨm, associated with the cellular uptake of the cationic dye Rhodamine123 and high fluorescence intensity. However, upon AD0157 treatment (1, 5, and 10 μM), cells revealed a reduction of Rhodamine123 incorporation, evidenced by a dose-dependent decrease of the relative fluorescence intensity, due to loss of ΔΨm (**Figure 6B**). This fact suggests that the drug provoked a drop in the cell mitochondrial membrane potential, this effect being more relevant in the AML cells.

**FIGURE 7:**
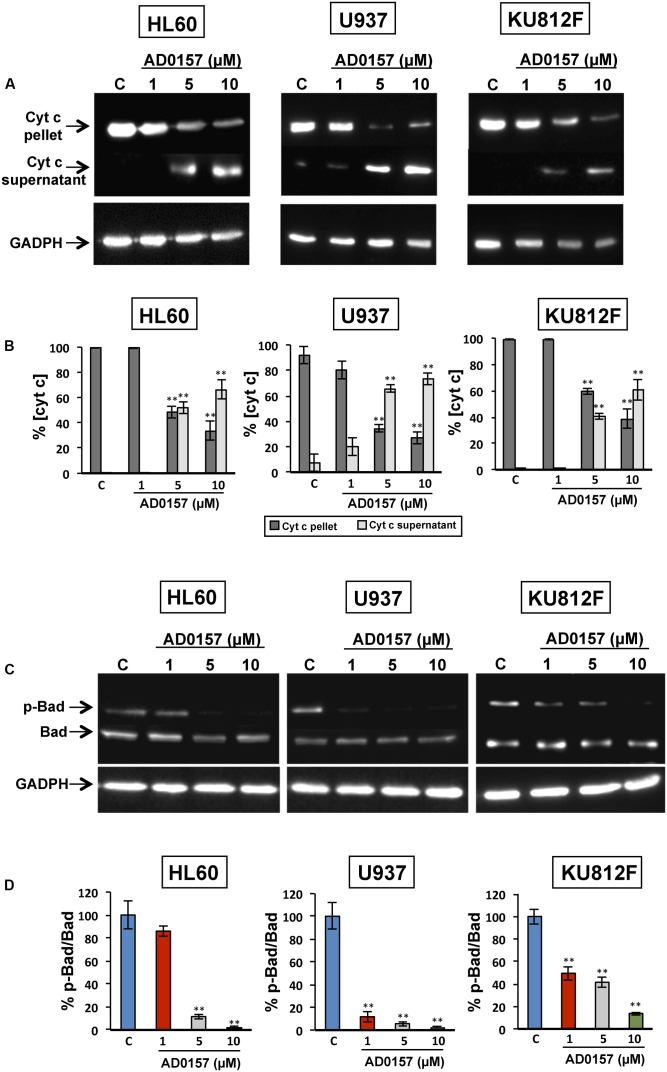
AD0157 induces the release of cytochrome c into the cytosol and suppresses Bad phosphorylation. **(A)** Detection of cytochrome c in the cytosolic (supernatant) and mitochondrial (pellet) fractions of AD0157-treated and non-treated leukemia cells. HL60, U937 and KU812F cells incubated with AD0157 were suspended in MSH buffer containing EGTA, succinate and MgCl_2,_ permeabilized with digitonin and the cytosolic and mitochondrial fractions were separated and subjected to Western blots. **(B)** Percentage of cytochrome c in the pellet (mitochondria) and supernatant (cytosol) after treatment with AD0157, expressed as means ± SD of three independent assays. *^∗∗^p* < 0.001 *versus* control. **(C)** Bad phosphorylation status in treated and untreated HL60, U937 and KU812 cells. Representative blots of p-Bad and total Bad in the studied leukemia cell lines. **(D)** Percentage of p-Bad/Bad ratios expressed as means ± SD of three independent assays. *^∗∗^p* < 0.001 *versus* control.

The changes in the physiology of the mitochondria after incubation with AD0157 (1, 5, and 10 μM) were also studied through the analysis of mitochondrial-related apoptotic markers such as cytochrome c, p-Bad and Bad proteins. In the intrinsic or mitochondrial apoptotic pathway the release of cytochrome c into the cytosol facilitates the apoptosome assembly, crucial for the activation of procaspase-9. Indeed, treatment of human leukemia cells with AD0157 caused the release of cytochrome c from mitochondria to cytosol in a dose-response fashion (**Figure 7A**). Moreover, a significant decrease of mitochondrial cytochrome c (cyt c in pellet) and a parallel increase of cytosolic cytochrome c (cyt c in supernatant) were observed from 5 μM of the compound (**Figure 7B**).

In order to further confirm the involvement of mitochondria in the AD0157-induced leukemia cell apoptosis, the levels of Bad and p-Bad were examined. Bad is a pro-apoptotic member of the BCL-2 family and it is regulated by phosphorylation-dephosphorylation in response to extracellular stimuli. As expected, Bad phosphorylation was prevented by AD0157 in the three cell lines studied, with a more remarkable effect on both AML cell lines (**Figures 7C,D**).

### Akt and ERK Regulate AD0157-Induced Apoptosis in Human Myeloid Leukemia Cells

**FIGURE 8:**
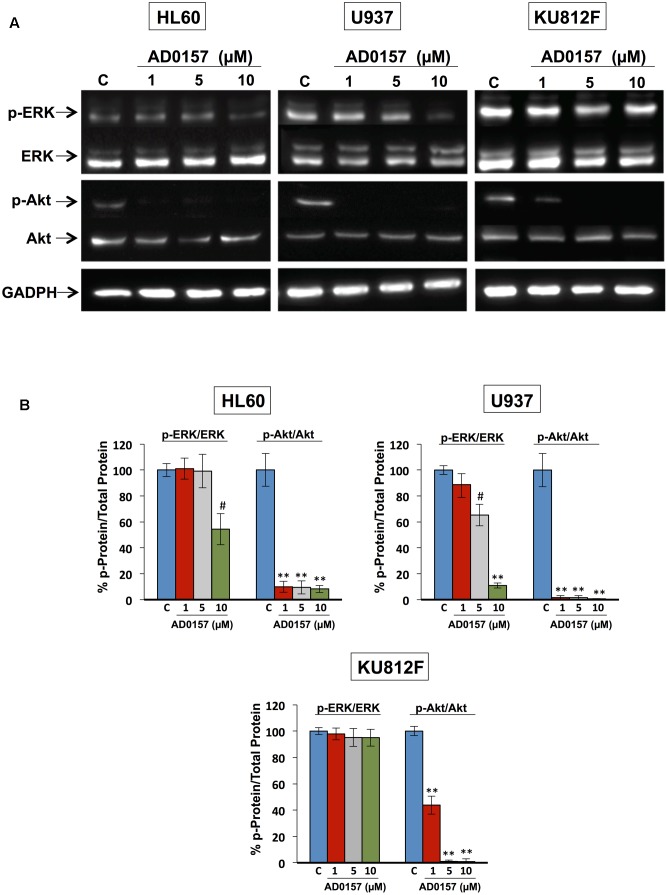
Akt and ERK regulate the AD0157-induced apoptosis in human myeloid leukemia cells. **(A)** Representative blots of p-ERK, total ERK, p-Akt and total Akt in HL60, U937 and KU812F upon treatment (14 h) with the indicated concentrations of AD0157 and stimulation with FBS 20% (30 min). **(B)** Percentage of p-ERK/ERK and p-Akt/Akt ratios in untreated and treated cells, expressed as means ± SD of three independent assays. ^#^*p* < 0.005 and ^∗∗^*p* < 0.001 *versus* control.

The phosphatidylinositol-3 Kinase/protein kinase B (PI3K/Akt) and mitogen-activated protein kinase (MAPK/ERK) signaling pathways, among others, are frequently activated in a wide variety of human cancers, including leukemia, and other hematopoietic disorders. PI3K/Akt cascade is involved in mediating survival and apoptosis signals, and MAPK/ERK is essential for the regulation of multiple key cellular functions including cell growth, proliferation, differentiation and migration. Therefore, we next conducted a mechanistic exploration to provide evidence about the effect of this compound on the phosphorylation of ERK1/2 and Akt proteins. For these studies, human leukemia cells were incubated with different doses of AD0157 (1, 5, and 10 μM) and then, stimulated with serum. As represented in **Figures 8A,B**, the impact of AD0157 in Akt phosphorylation was significantly abrogated from 1 μM AD0157 in AML and CML cell lines. Indeed, the phosphorylation levels were reduced more than 80% at only 1 μM of compound in AML cells, and at 5 μM in CML cells. In contrast, analysis of ERK phosphorylation status at the same doses, revealed no phosphorylation repression at 1 and 5 μM AD0157 (**Figures 8A,B**). When increasing AD0157 concentration up to 10 μM a significant inhibition in the phosphorylated-ERK levels was observed in both AML cell lines, but not in the CML cell line (**Figures 8A,B**). Together, these data suggest that the Akt survival pathway may be a major target for AD0157 in leukemia cells, the effect of this compound being less relevant on the MAPK/ERK1/2 signaling cascade.

## Discussion

The development of new drugs that are able to trigger apoptosis in aggressive hematological malignancies such as AML and CML, restoring their sensitivity to apoptosis stimuli, can be considered a promising antileukemic strategy. In this work we demonstrate that AD0157, in the low micromolar range, inhibits the growth of two AML cell lines and a CML cell line characterized by the BCR-ABL fused protein. Interestingly, this effect presents a relative grade of selectivity, since it is exerted at concentrations that do not affect the growth of other human tumor cell lines, including fibrosarcoma, osteosarcoma, breast carcinoma and colon adenocarcinoma (García-Caballero et al., 2014). Moreover, the inhibitory effect of this compound on the growth of non-transformed cells was also exhibited at higher concentrations to those required for leukemia cells, as evidenced by comparison of the IC_50_ values obtained by MTT assay with that reported for a primary culture of endothelial cells (García-Caballero et al., 2014).

The finding that the antiangiogenic activity of AD0157 could be mediated, at least in part, by induction of endothelial cell apoptosis brought us to postulate a putative apoptogenic activity of this compound on myeloid leukemia cells as being responsible for the strong growth inhibitory activity observed in the MTT reduction assay. Firstly, a pilot study with different AD0157 concentrations ranging from 0.5 to 15 μM was carried out to analyze apoptosis induction in human leukemia cells upon 14 h of treatment. Then, three of these doses were considered as the most adequate concentrations to show a dose-response effect by AD0157, and replicates were performed. Different approaches, with different sensitivity of detection, were used in our experimental battery. Thus, induction of apoptosis in the three cell lines was initially suggested by the presence of nuclei with condensed chromatin and DNA fragmentation, assessed by Hoechst staining and labeling of DNA strand breaks with fluorochrome-tagged deoxynucleotides by exogenous terminal deoxynucleotidyl transferase (TUNEL), respectively. The proapoptotic activity of AD0157 on leukemia cells was further confirmed by a significant increase in the subG1 subpopulation, containing subdiploid or apoptotic cells, as demonstrated by flow cytometry after propidium iodide staining. Surprisingly, this effect was obtained at concentrations that were at least five-fold lower than those required for normal endothelial cells (García-Caballero et al., 2014). Whereas healthy cells are characterized by the asymmetric distribution of plasma membrane phospholipids between inner and outer sides, during apoptosis phosphatidylserine becomes exposed on the outside face of the membrane, attracting macrophages, which will phagocytize apoptotic cells and apoptotic bodies (Fadok et al., 1992). The induction of early apoptosis by AD0157 was confirmed by the observation that at concentrations of 1 μM or higher of drug, the translocation of phosphatidylserine from the inner face of the phospholipid bilayer to the cell surface was provoked.

The crucial role of mitochondria in the regulation of apoptosis is widely accepted, and this fact makes them an attractive target for the development of new antitumor drugs (Fulda and Kroemer, 2011). In the present study, we provide evidence that AD0157 induces mitochondrial membrane perturbation, by triggering a dissipation of the mitochondrial transmembrane potential and causing the release of pro-apoptotic proteins such as cytochrome c from the mitochondrial intermembrane space into the cytosol. This suggests that mitochondria stands at the nexus of sensing and integrating the stress caused by this compound in myeloid leukemia cells. Mitochondrial disturbances often occur long before any marked morphological symptoms of apoptosis. Dissipation of mitochondrial transmembrane electrochemical potential (ΔΨm) has been marked by some authors as the “point of no return” along the apoptotic pathway. Although the correlation of the dissipation of ΔΨm and late apoptotic events remains controversial (Li et al., 2000), the cytometric detection of ΔΨm loss is acknowledged as a sensitive marker of early apoptotic events (Wlodkowic et al., 2011).

To gain greater insight into the mechanisms involved in the induction of apoptosis by AD0157, caspase activities were studied. Our results show that AD0157 simultaneously increases the activity of the initiator caspases-8 and -9, as well as the downstream activation of the executioner caspases-3 and -7, acting during the late steps of the apoptotic process. The caspase inhibitor Z-VAD-FMK completely reversed the apoptogenic activity of AD0157, suggesting this to be mediated by caspase-dependent mechanisms.

**FIGURE 9:**
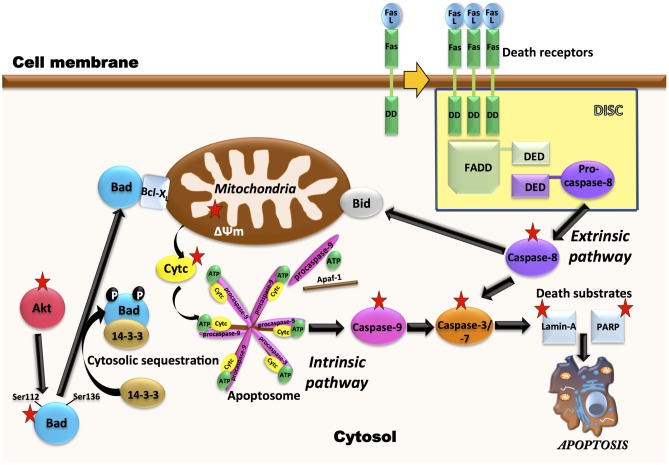
Multiple molecular targets are simultaneously involved in the pro-apoptotic effect of AD0157 on human myeloid leukemia cells. On the one hand, in the control of intrinsic pathway of apoptosis, inhibition of Akt phosphorylation by AD0157 could lead to dephosphorylation of pro-apoptotic Bad, causing a drop in the mitochondrial membrane potential and triggering the release of cytochrome c into the cytosol. This could facilitate the formation of apoptosome, which activates the initiator caspase-9 that would cause an activation of executioner caspases-3/-7, ultimately leading to apoptosis. On the other hand, the effect of AD0157 on the extrinsic pathway of apoptosis is suggested by the increase in the caspase-8 activity, which could then prompt the activation of the effector caspases. In addition, caspase-8 could initiate a mitochondrial amplification loop, mediated by the cleavage of the BH3-only protein Bid. In the scheme, targets of AD0157 are indicated with a red star.

Leukemia cells that escape from extrinsic apoptotic pathways, may acquire chemotherapy resistance, so the development of new antileukemic therapeutic strategies based on the induction of apoptosis is of growing interest (Al-Hussaini and DiPersio, 2014; Fan et al., 2015). Our results, indicating activation of caspase-8 by AD0157, as well as a decrease in the toxicity of this compound in the presence of a caspase-8 inhibitor, although preliminary, suggest that activation of the extrinsic pathway of apoptosis could be related to the AD0157 death-inducing activity on leukemia cells (**Figure 9**). On the other hand, mitochondrial (intrinsic) pathway of apoptosis is controlled by proteins of Bcl2 family, such as the pro-apoptotic Bad protein (Zha et al., 1996). The pro-apoptotic protein Bad is an upstream sensor of cellular damage that is regulated by a phosphorylation/dephosphorylation mechanism in response to extracellular stimuli (Delbridge and Strasser, 2015). While phosphorylation of Bad in serine residues promotes its association with the scaffold protein 14-3-3 (sequester Bad in cytosol) and protects cells from apoptosis, Bad dephosphorylation induces its heterodymerization with Bcl-XL in the outer mitochondrial membrane, which provokes changes in the mitochondria permeability and triggers in favor of apoptosis (Yang et al., 1995). Therefore, dephosphorylated Bad promotes cytochrome c release to cytosol, where it interacts with the apoptotic protease activating factor 1 (Apaf-1), procaspase-9 and dATP to form the apoptosome (Reubold and Eschenburg, 2012). After being activated by apoptosome, caspase-9 activates downstream effector caspases involved in the cleavage of substrates, which are directly related to the apoptotic response (Reubold and Eschenburg, 2012). Results shown here clearly demonstrate that AD0157 induces apoptosis in myeloid leukemia cells by activation of the intrinsic pathway (**Figure 9**). They include Bad dephosphorylation, cytochrome c release from mitochondria, initiator caspase-9 and executioner caspase-3 and -7 activation. Furthermore, we observe the cleavage of death substrates as lamin-A, a structural protein belonging to the intermediate filament family essential for the scaffolding of the nuclear envelope, and PARP, a nuclear enzyme involved in DNA repair that is activated in response to DNA damage. It is also worth mentioning that the addition of a caspase-9 inhibitor clearly compromised the apoptogenic activity of AD0157 on the myeloid leukemia cells.

The constitutive activation of some signaling pathways, including PI3K/Akt and MAPK/ERK, controlling cell growth, survival and apoptosis, has been implicated in both the pathogenesis and the progression of myeloid leukemias (Testa and Riccioni, 2007). PI3K/Akt signaling is frequently activated in AML patient blasts and strongly contributes to proliferation, survival and drug resistance of these cells (Martelli et al., 2006). MAPK/ERK signaling pathway is constitutively activated in the majority of AML patients (Daver and Cortes, 2012). Alternatively, BCR-ABL fused protein, characteristic of CML, activates Akt and ERK pathways, which leads to deregulated growth and resistance to apoptosis (Hazlehurst et al., 2009; Quintas-Cardama and Cortes, 2009). According to data presented here, AD0157 strongly inhibits the phosphorylation of Akt in the different human leukemia cells studied. Given the crucial roles played by the activation of Akt-dependent signaling cascades in the development of many tumor types and resistance to chemotherapy, the ability of AD0157 to interfere with Akt phosphorylation needs further additional evaluation and analyses on other tumor cell types are essential. A significant inhibition of ERK phosphorylation only detected in AML cells at higher concentrations of this compound, reveals a less relevant role of this pathway, if any, in the antileukemic mechanism of action of this compound. This is in agreement with the results arising from cell cycle studies showing that upon 14 h of AD0157 treatment, this compound induces cell death whereas no inhibition of proliferation, reflected by a blockade in some of the cell cycle checkpoints, was detected. Since Akt and ERK pathways have been reported to regulate the phosphorylation state of Bad (Scheid et al., 1999), inactivation of any (or both) pathways could render the observed decrease in Bad phosphorylation after incubation with AD0157 (**Figure 9**).

To summarize, in this investigation we present the induction of apoptosis in human myeloid leukemia cells by the natural marine compound AD0157. Despite being the first evidence of the potential of AD0157 as a new drug candidate for the treatment of myeloid leukemia, our results also give insight into the molecular mechanisms of this compound ending up in cell death, presented in **Figure 9**. The observation of pro-apoptotic effects in both AML and CML cells broadens the potential of AD0157 as an apoptosis-inducing compound in myeloid leukemia cells. Although pathological angiogenesis was initially recognized as a hallmark of solid tumors, recent studies indicate that angiogenesis is also important in the pathogenesis and progression of several forms of leukemia, including AML and CML (Molica et al., 2004; Trujillo et al., 2012; AbdElAal et al., 2015; Shirzad et al., 2016). The previously reported antiangiogenic activity of AD0157 altogether with data presented here, indicating a direct antileukemic effect of this compound by induction of apoptosis pathways, suggest that this natural compound could be a potential new agent for the treatment of myeloid leukemia. Further preclinical and clinical studies should verify this assessment.

## Author Contributions

MG-C, MM, and AQ designed the experiments, discussed the results and wrote the manuscript. MG-C and BM-P performed the experiments.

## Conflict of Interest Statement

The authors declare that the research was conducted in the absence of any commercial or financial relationships that could be construed as a potential conflict of interest.

## References

[B1] AbdElAal A. A. Afify R. A. Zaher A. E. ElGammal M. M. Atef A. M. (2015). Study of prognostic significance of marrow angiogenesis assessment in patients with de novo acute leukemia. *Hematology* 20 504–510. 10.1179/1607845415Y.0000000012 25885121

[B2] Al-Hussaini M. DiPersio J. F. (2014). Small molecule inhibitors in acute myeloid leukemia: from the bench to the clinic. *Expert Rev. Hematol.* 7 439–464. 10.1586/17474086.2014.932687 25025370 PMC4283573

[B3] Amirkia V. Heinrich M. (2015). Natural products and drug discovery: a survey of stakeholders in industry and academia. *Front. Pharmacol.* 6:237. 10.3389/fphar.2015.00237 26578954 PMC4620409

[B4] Baracca A. Sgarbi G. Solaini G. Lenaz G. (2003). Rhodamine 123 as a probe of mitochondrial membrane potential: evaluation of proton flux through F_0_ during ATP synthesis. *Biochim. Biophys. Acta* 1606 137–146. 10.1016/S0005-2728(03)00110-5 14507434

[B5] Blunt J. W. Copp B. R. Keyzers R. A. Munro M. H. Prinsep M. R. (2017). Marine natural products. *Nat. Prod. Rep.* 34 235–294. 10.1039/c6np00124f 28290569

[B6] Crowley L. C. Marfell B. J. Waterhouse N. J. (2016). Detection of DNA fragmentation in apoptotic cells by TUNEL. *Cold Spring Harb. Protoc.* 2016 900–905. 10.1101/pdb.prot087221 27698233

[B7] Daver N. Cortes J. (2012). Molecular targeted therapy in acute myeloid leukemia. *Hematology* 17(Suppl. 1), S59–S62. 10.1179/102453312X13336169155619 22507781

[B8] Delbridge A. R. Strasser A. (2015). The BCL-2 protein family, BH3-mimetics and cancer therapy. *Cell Death Differ.* 22 1071–1080. 10.1038/cdd.2015.50 25952548 PMC4572872

[B9] Dombret H. Gardin C. (2016). An update of current treatments for adult acute myeloid leukemia. *Blood* 127 53–61. 10.1182/blood-2015-08-604520 26660429 PMC4705610

[B10] Fadok V. A. Voelker D. R. Campbell P. A. Cohen J. J. Bratton D. L. Henson P. M. (1992). Exposure of phosphatidylserine on the surface of apoptotic lymphocytes triggers specific recognition and removal by macrophages. *J. Immunol.* 148 22–29. 1545126

[B11] Fan D. Li Z. Zhang X. Yang Y. Yuan X. Zhang X. (2015). AntiCD3Fv fused to human interleukin-3 deletion variant redirected T cells against human acute myeloid leukemic stem cells. *J. Hematol. Oncol.* 8:18. 10.1186/s13045-015-0109-5 25879549 PMC4389834

[B12] Fukuda T. Kishi K. Ohnishi Y. Shibata A. (1987). Bipotential cell differentiation of KU-812: evidence of a hybrid cell line that differentiates into basophils and macrophage-like cells. *Blood* 70 612–619. 3304457

[B13] Fulda S. (2009). Cell death in hematological tumors. *Apoptosis* 14 409–423. 10.1007/s10495-008-0306-6 19130230

[B14] Fulda S. Kroemer G. (2011). Mitochondria as therapeutic targets for the treatment of malignant disease. *Antioxid. Redox Signal.* 15 2937–2949. 10.1089/ars.2011.4078 21644835

[B15] García-Caballero M. Cañedo L. Fernández-Medarde A. Medina M. A. Quesada A. R. (2014). The marine fungal metabolite, AD0157, inhibits angiogenesis by targeting the Akt signaling pathway. *Mar. Drugs* 12 279–299. 10.3390/md12010279 24441613 PMC3917274

[B16] García-Caballero M. Marí-Beffa M. Cañedo L. Medina M. Á. Quesada A. R. (2013). Toluquinol, a marine fungus metabolite, is a new angiosuppresor that interferes with the Akt pathway. *Biochem. Pharmacol.* 85 1727–1740. 10.1016/j.bcp.2013.04.007 23603293

[B17] García-Caballero M. Marí-Beffa M. Medina M. Á. Quesada A. R. (2011). Dimethylfumarate inhibits angiogenesis in vitro and in vivo: a possible role for its antipsoriatic effect? *J. Invest. Dermatol.* 131 1347–1355. 10.1038/jid.2010.416 21289642

[B18] Gruenbaum Y. Wilson K. L. Harel A. Goldberg M. Cohen M. (2000). Review: nuclear lamins- structural proteins with fundamental functions. *J. Struct. Biol.* 129 313–323. 10.1006/jsbi.2000.4216 10806082

[B19] Hanahan D. Weinberg R. A. (2011). Hallmarks of cancer: the next generation. *Cell* 144 646–674. 10.1016/j.cell.2011.02.013 21376230

[B20] Hassan M. Watari H. AbuAlmaaty A. Ohba Y. Sakuragi N. (2014). Apoptosis and molecular targeting therapy in cancer. *Biomed Res. Int.* 2014:150845. 10.1155/2014/150845 25013758 PMC4075070

[B21] Hazlehurst L. A. Bewry N. N. Nair R. R. Pinilla-Ibarz J. (2009). Signaling networks associated with BCR-ABL-dependent transformation. *Cancer Control* 16 100–107. 10.1177/107327480901600202 19337196

[B22] Hui K. K. Kanungo A. K. Elia A. J. Henderson J. T. (2011). Caspase-3 deficiency reveals a physiologic role for Smac/DIABLO in regulating programmed cell death. *Cell Death Differ.* 18 1780–1790. 10.1038/cdd.2011.50 21597464 PMC3190114

[B23] Itzykson R. Duchmann M. Lucas N. Solary E. (2017). CMML: clinical and molecular aspects. *Int. J. Hematol.* 105 711–719. 10.1007/s12185-017-2243-z 28455647

[B24] Kim R. Emi M. Tanabe K. (2006). The role of apoptosis in cancer cell survival and therapeutic outcome. *Cancer Biol. Ther.* 5 1429–1442. 10.4161/cbt.5.11.3456 17102590

[B25] Kinghorn A. D. Chin Y. W. Swanson S. (2010). Discovery of natural product anticancer agents from biodiverse organisms. *Curr. Opin. Drug Discov. Dev.* 12 189–196. 19333864 PMC2877274

[B26] Koeffler H. P. Golde D. W. (1980). Human myeloid leukemia cell lines: a review. *Blood* 56 344–350. 6996765

[B27] Larrick J. W. Fischer D. G. Anderson S. J. Koren H. S. (1980). Characterization of a human macrophage-like cell line stimulated in vitro: a model of macrophage functions. *J. Immunol.* 125 6–12. 7381211

[B28] Lee E. W. Seo J. Jeong M. Lee S. Song J. (2012). The roles of FADD in extrinsic apoptosis and necroptosis. *BMB Rep.* 45 496–508. 10.5483/BMBRep.2012.45.9.186 23010170

[B29] Li X. Du L. Darzynkiewicz Z. (2000). During apoptosis of HL-60 and U-937 cells caspases are activated independently of dissipation of mitochondrial electrochemical potential. *Exp. Cell Res.* 257 290–297. 10.1006/excr.2000.4901 10837143

[B30] Liersch R. Müller-Tidow C. Berdel W. E. Krug U. (2014). Prognostic factors for acute myeloid leukaemia in adults–biological significance and clinical use. *Br. J. Haematol.* 165 17–38. 10.1111/bjh.12750 24484469

[B31] Lück S. C. Russ A. C. Botzenhardt U. Paschka P. Schlenk R. F. Döhner H. (2011). Deregulated apoptosis signaling in core-binding factor leukemia differentiates clinically relevant, molecular marker-independent subgroups. *Leukemia* 25 1728–1738. 10.1038/leu.2011.154 21701487

[B32] Malve H. (2016). Exploring the ocean for new drug developments: marine pharmacology. *J. Pharm. Bioallied Sci.* 8 83–91. 10.4103/0975-7406.171700 27134458 PMC4832911

[B33] Malvezzi M. Carioli G. Bertuccio P. Rosso T. Boffetta P. Levi F. (2016). European cancer mortality predictions for the year 2016 with focus on leukaemias. *Ann. Oncol.* 27 725–731. 10.1093/annonc/mdw022 26812903

[B34] Man M. S. Kanneganti T. D. (2016). Converging roles of caspases in inflammasome activation, cell death and innate immunity. *Nat. Rev. Immunol.* 16 7–21. 10.1038/nri.2015.7 26655628 PMC4915362

[B35] Martelli A. M. Nyåkern M. Tabellini G. Bortul R. Tazzari P. L. Evangelisti C. (2006). Phosphoinositide 3-kinase/Akt signaling pathway and its therapeutical implications for human acute myeloid leukemia. *Leukemia* 20 911–928. 10.1038/sj.leu.2404245 16642045

[B36] Martínez-Poveda B. Rodríguez-Nieto S. García-Caballero M. Medina M. Á. Quesada A. R. (2012). The antiangiogenic compound aeroplysinin-1 induces apoptosis in endothelial cells by activating the mitochondrial pathway. *Mar. Drugs* 10 2033–2046. 10.3390/md10092033 23118719 PMC3475271

[B37] Molica S. Vacca A. Levato D. Merchionne E. Ribatti D. (2004). Angiogenesis in acute and chronic lymphocytic leukemia. *Leuk. Res.* 28 1239–1240. 10.1016/j.leukres.2003.08.001 15109528

[B38] Pistritto G. Trisciuoglio D. Ceci C. Garufi A. D’Orazi G. (2016). Apoptosis as anticancer mechanism: function and dysfunction of its modulators and targeted therapeutic strategies. *Aging* 8 603–619. 10.18632/aging.100934 27019364 PMC4925817

[B39] Pradelli L. A. Bénéteau M. Ricci J. E. (2010). Mitochondrial control of caspase-dependent and -independent cell death. *Cell Mol. Life Sci.* 67 1589–1597. 10.1007/s00018-010-0285-y 20151314 PMC11115767

[B40] Quintas-Cardama A. Cortes J. (2009). Molecular biology of bcr-abl1-positive chronic myeloid leukemia. *Blood* 113 1619–1630. 10.1182/blood-2008-03-144790 18827185 PMC3952549

[B41] Reed J. C. Pellecchia M. (2005). Apoptosis-based therapies for hematologic malignancies. *Blood* 106 408–418. 10.1182/blood-2004-07-2761 15797997

[B42] Reubold T. F. Eschenburg S. (2012). A molecular view on signal transduction by the apoptosome. *Cell. Signal.* 24 1420–1425. 10.1016/j.cellsig.2012.03.007 22446004

[B43] Satoh M. S. Lindahl T. (1992). Role of poly(ADP-ribose) formation in DNA repair. *Nature* 356 356–358. 10.1038/356356a0 1549180

[B44] Saygin C. Carraway H. E. (2017). Emerging therapies for acute myeloid leukemia. *J. Hematol. Oncol.* 10 93. 10.1186/s13045-017-0463-6 28420416 PMC5395764

[B45] Scheid M. P. Schubert K. M. Duronio V. (1999). Regulation of bad phosphorylation and association with Bcl-x_L_ by the MAPK/Erk kinase. *J. Biol. Chem.* 274 31108–31113. 10.1074/jbc.274.43.31108 10521512

[B46] Shirzad R. Shahrabi S. Ahmadzadeh A. Kampen K. R. Shahjahani M. Saki N. (2016). Signaling and molecular basis of bone marrow niche angiogenesis in leukemia. *Clin. Transl. Oncol.* 18 957–971. 10.1007/s12094-015-1477-6 26742939

[B47] Siegel R. L. Miller K. D. Jemal A. (2017). Cancer statistics, 2017. *CA Cancer J. Clin.* 67 7–30. 10.3322/caac.21387 28055103

[B48] Sun S. Y. Hail N. Jr. Lotan R. (2004). Apoptosis as a novel target for cancer chemoprevention. *J. Natl. Cancer Inst.* 96 662–672. 10.1093/jnci/djh123 15126603

[B49] Testa U. Riccioni R. (2007). Deregulation of apoptosis in acute myeloid leukemia. *Haematologica* 92 81–89. 10.3324/haematol.10279 17229639

[B50] Trujillo A. McGee C. Cogle C. R. (2012). Angiogenesis in acute myeloid leukemia and opportunities for novel therapies. *J. Oncol.* 2012:128608. 10.1155/2012/128608 21904549 PMC3167188

[B51] Vanden Berghe T. Linkermann A. Jouan-Lanhouet S. Walczak H. Vandenabeele P. (2014). Regulated necrosis: the expanding network of non-apoptotic cell death pathways. *Nat. Rev. Mol. Cell Biol.* 15 135–147. 10.1038/nrm3737 24452471

[B52] Vaux D. L. Korsmeyer S. J. (1999). Cell death in development. *Cell* 96 245–254. 10.1016/S0092-8674(00)80564-4s 9988219

[B53] Vermeulen K. Van Bockstaele D. R. Berneman Z. N. (2005). Apoptosis: mechanisms and relevance in cancer. *Ann. Hematol.* 84 627–639. 10.1007/s00277-005-1065-x 16041532

[B54] Vogler M. Walter H. S. Dyer M. J. S. (2017). Targeting anti-apoptotic BCL2 family proteins in haematological malignancies - from pathogenesis to treatment. *Br. J. Haematol.* 178 364–379. 10.1111/bjh.14684 28449207

[B55] Wlodkowic D. Telford W. Skommer J. Darzynkiewicz Z. (2011). Apoptosis and beyond: cytometry in studies of programmed cell death. *Methods Cell Biol.* 103 55–98. 10.1016/B978-0-12-385493-3.00004-8 21722800 PMC3263828

[B56] Yang E. Zha J. Jockel J. Boise L. H. Thompson C. B. Korsmeyer S. J. (1995). Bad, a heterodimeric partner for Bcl-XL and Bcl-2, displaces Bax and promotes cell death. *Cell* 80 285–291. 10.1016/0092-8674(95)90411-5 7834748

[B57] Zha J. Harada H. Yang E. Jockel J. Korsmeyer S. J. (1996). Serine phosphorylation of death agonist BAD in response to survival factor results in binding to 14-3-3 not BCL-X_L_. *Cell* 87 619–628. 10.1016/S0092-8674(00)81382-3 8929531

